# Crystal structure of 5-chloro-*N* ^1^-(5-phenyl-1*H*-pyrazol-3-yl)benzene-1,2-di­amine

**DOI:** 10.1107/S2056989017007381

**Published:** 2017-05-26

**Authors:** Yegor Yartsev, Vitaliy Palchikov, Alexandr Gaponov, Svitlana Shishkina

**Affiliations:** a V. N. Karazin Kharkiv National University, 4 Svobody Sq, Kharkiv 61077, Ukraine; b Oles Honchar Dnipropetrovsk National University, 72 Gagarina St, Dnipropetrovsk 49010, Ukraine; c SSI "Institute for Single Crystals", National Academy of Sciences of Ukraine, 60 Nauky Ave., Kharkiv 61001, Ukraine

**Keywords:** crystal structure, pyrazol-3-amine, steric repulsion, hydrogen bonding

## Abstract

The title compound, C_15_H_13_ClN_4_, crystallizes with two independent mol­ecules (*A* and *B*) in the asymmetric unit, which are far from planar as a result of steric repulsion between the rings. The benzene and phenyl rings are inclined to the central pyrazole ring by 46.64 (10) and 17.87 (10)° in mol­ecule *A*, and by 40.02 (10) and 14.18 (10)° in mol­ecule *B*. The aromatic rings are inclined to one another by 58.77 (9)° in mol­ecule *A*, and 36.95 (8)° in mol­ecule *B*. In the crystal, the *A* and *B* mol­ecules are linked by two pairs of N—H⋯N hydrogen bonds forming *A*–*B* dimers. These are further linked by a fifth N—H⋯N hydrogen bond, forming tetra­mer-like units that stack along the *a*-axis direction, forming columns, which are in turn linked by C—H⋯π inter­actions, forming layers parallel to the *ac* plane.

## Abstract 2

The title compound, crystallizes with two independent mol­ecules (*A* and *B*) in the asymmetric unit, which are far from planar. The aryl rings are inclined to one another by 58.77 (9)° in mol­ecule *A* and by 36.95 (8)° in mol­ecule *B*.

## Chemical context   

**Figure 1:**
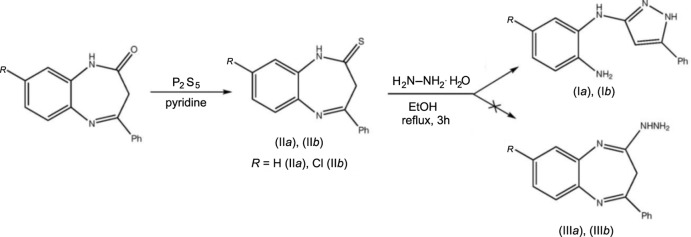
Synthesis scheme for the title compound (I*b*).

The synthesis and reactions of benzodiazepin-2-ones and thio­nes have been studied in detail by our group (Gaponov *et al.*, 2016 ▸; Okovytyy *et al.*, 2009 ▸). The mechanism of ethanol-assisted hydrazinolysis of 1,3-di­hydro-2*H*-benzo[*b*][1,4]diazepine-2-thio­nes (Fig. 1 ▸) has been modelled by quantum-chemical calculations (Okovytyy *et al.*, 2009 ▸). However, instead of obtaining the previously suggested products (III*a*) and (III*b*), compounds *N* ^1^-(5-phenyl-1*H*-pyrazol-3-yl)benzene-1,2-di­amine (I*a*) and its 5-chloro-derivative (I*b*) were prepared from 4-phenyl-1,3-di­hydro-2*H*-benzo[*b*][1,4]diazepine-2-thio­nes (II*a*) and (II*b*) and hydrazine hydrate (Fig. 1 ▸). Amino­pirazoles are useful building blocks for the synthesis of new pharmaceutical agents (Sakya *et al.*, 2006 ▸) and agrochemicals (Yuan *et al.*, 2013 ▸), due to their notable biological properties (Peng *et al.*, 2013 ▸; Zhang *et al.*, 2014 ▸; Ansari *et al.*, 2017 ▸). The crystal structure analysis of the title compound, (I*b*), was undertaken as it may help to provide a better understanding of the properties of amino­pirazoles.

## Structural commentary   

**Figure 2:**
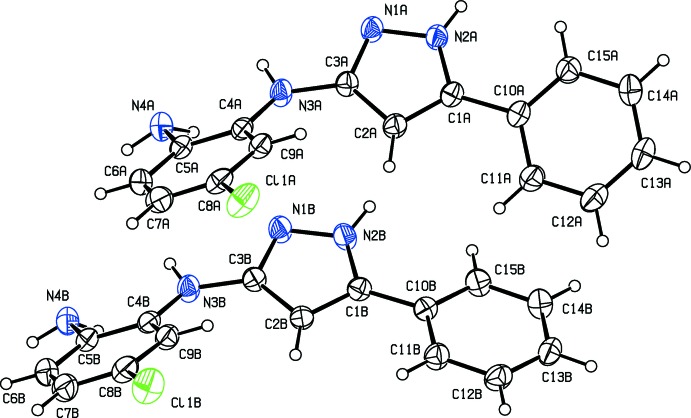
The mol­ecular structure of the two independent mol­ecules (*A* and *B*) of compound (I*b*), with the atom labelling. Displacement ellipsoids are drawn at the 30% probability level.

There are two independent mol­ecules (*A* and *B*) in the asymmetric unit of the title compound (I*b*), as illustrated in Fig. 2 ▸. They are composed of three unsaturated rings, two of which are connected by a bridging amino group. The mol­ecules are not planar as a result of steric repulsion between the rings, which results in some disturbance of the conjugation. Thus, the presence of a shortened intra­molecular contact C2 ⋯ H11 [2.80 Å in mol­ecule *A* and 2.81 Å in mol­ecule *B* as compared with the sum of their van der Waals radii of 2.87 Å (Zefirov, 1997 ▸)], indicates the presence of repulsion between the pyrazole ring and the phenyl substituent. The steric strain is compensated for by the elongation of the C1—C10 bond: 1.486 (2) Å in mol­ecule *A* and 1.482 (2) Å in mol­ecule *B* compared to a mean bond length of 1.470 Å for a typical conjugated system (Bürgi & Dunitz, 1994 ▸). In addition, the C2—C1—C10 bond angle increases to 130.6 (2)° in both mol­ecules, and the pyrazole and phenyl rings are twisted with respect to each other, with torsion angle C2—C1—C10—C11 being 18.1 (3)° in mol­ecule *A* and −14.3 (3)° in mol­ecule *B*.
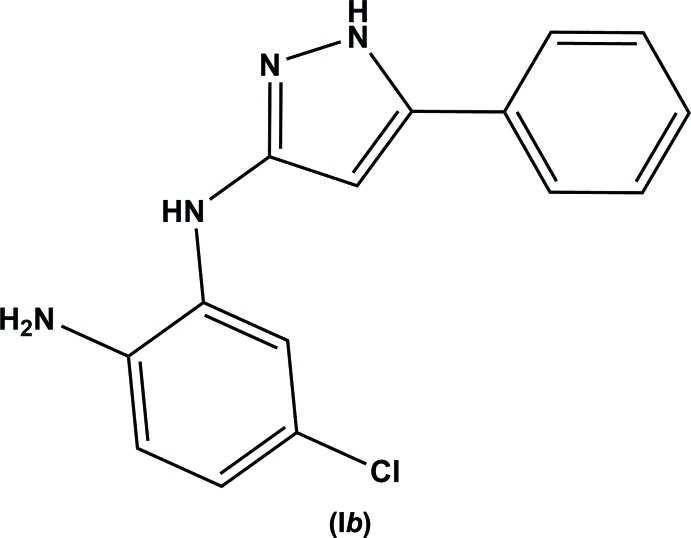


There is an even stronger repulsion between the amino­chloro­phenyl and pyrazole rings linked through the bridging amino group [shortened intra­molecular contacts are: C2⋯C9 = 3.25 Å (*A*), 3.21 Å (*B*); C2⋯H9 = 2.75 Å (*A*), 2.67 Å (*B*); H3⋯H4 = 2.28 Å for both mol­ecules; C3⋯H9 = 2.76 Å for both mol­ecules] leads to a greater twist of these unsaturated rings relative to each other; the dihedral angle between the mean planes N1/N2/C1–C3 and C4–C9 is 46.6 (1)° for mol­ecule *A* and 40.0 (1)° for *B*. Moreover, the N3—C3 bonds [1.395 (3) Å in *A* and 1.394 (2) Å in *B*; mean value of 1.339 Å] and the N3—C4 bonds [1.408 (2) Å in *A*, 1.406 (2) Å in *B*; mean value of 1.353 Å] are elongated with respect to the mean values for such bonds, and the C2=C3—N3 bond angle is increased to 130.3 (2)° in *A* and 130.5 (2)° in *B*.

The bridging nitro­gen atom, N3, has an almost planar configuration (the bond-angle sum is 356° in *A* and 358° in *B*). The N4H_2_ amino group has a pyramidal configuration (bond-angle sum is 329° in *A* and 325° in *B*). The C5—N4 bond, 1.422 (3) Å in *A* and 1.425 (3) Å in *B*, is elongated in comparison with the mean value of 1.394 Å; this elongation is probably caused by the involvement of the nitro­gen lone pair in hydrogen bonding (Table 1 ▸).

## Supra­molecular features   

**Figure 3:**
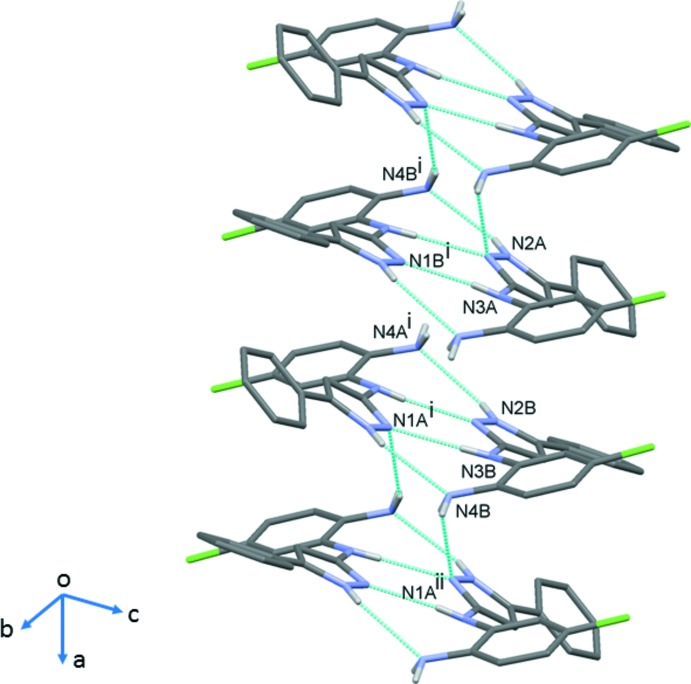
A view of the hydrogen-bonded (dashed lines; see Table 1 ▸) tetra­meric units of compound (I*b*). For clarity, only H atoms involved in hydrogen bonding have been included.

**Figure 4:**
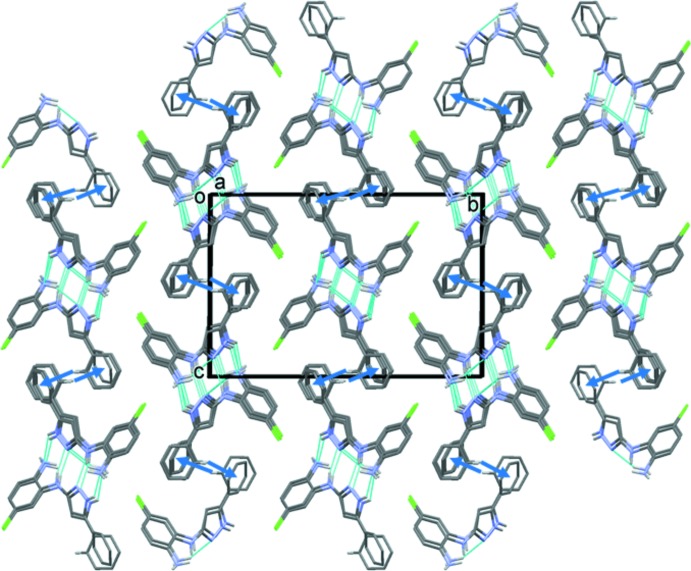
A view along the *a* axis of the crystal packing of compound (I*b*). The N—H⋯N hydrogen bonds are shown as dashed lines and the C—H⋯π inter­actions as blue arrows (see Table 1 ▸). For clarity, only the H atoms involved in these inter­actions have been included.

In the crystal, mol­ecules are linked by two pairs of N—H⋯N hydrogen bonds, forming *A*–*B* dimers (Table 1 ▸ and Fig. 3 ▸). The dimers are linked by a fifth N—H⋯N hydrogen bond to form a tetra­mer-like arrangement (Table 1 ▸ and Fig. 3 ▸). These stack up the *a*-axis direction, forming columns (Table 2 ▸ and Fig. 4 ▸), which are linked by C—H⋯π inter­actions, forming layers parallel to the *ac* plane.

## Database survey   

**Figure 5:**
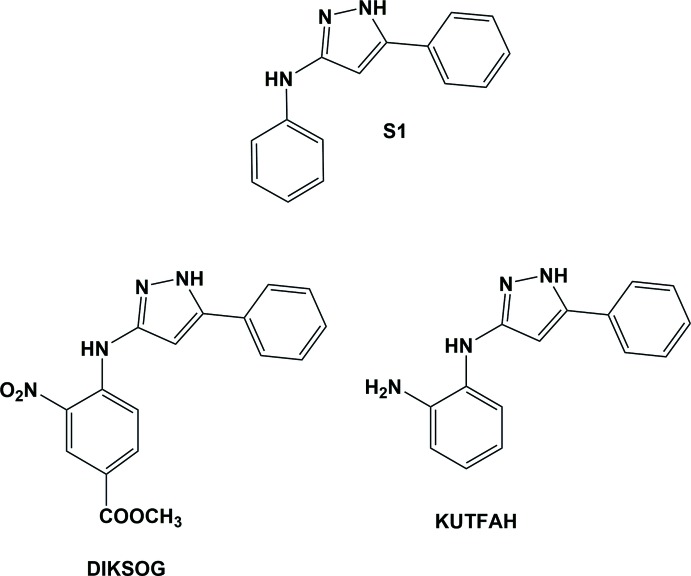
CSD search substructure S1, and relevant hits, KUTFAH and DIKSOG.

A search of the Cambridge Structural Database (Version 5.38, update February 2017; Groom *et al.*, 2016 ▸) for *N*,5-diphenyl-1*H*-pyrazol-3-amine (S1; Fig. 5 ▸) gave only two relevant hits, *viz.* methyl 3-nitro-4-[(5-phenyl-1*H*-pyrazol-3-yl)amino]­benzo­ate (DIKSOG; Portilla *et al.*, 2007 ▸) and *N*-(5-phenyl-1*H*-pyrazol-3-yl)benzene-1,2-di­amine (KUTFAH; Doumbia *et al.*, 2010 ▸). They differ from compound (I*b*) in the substituents on one of the aromatic rings (see Fig. 5 ▸). The mol­ecule of DIKSOG is practically planar, probably owing to the formation of intra­molecular N—H⋯O and C—H⋯N hydrogen bonds. In compound KUTFAH, while the phenyl ring is almost coplanar with the pyrazole ring (dihedral angle is *ca* 3.68° *cf.* 2.15° in DIKSOG), the *o*-amino­phenyl ring is inclined to the pyrazole ring by *ca* 64.03° (*cf.* 5.61° in DIKSOG). This conformation is similar to that of compound (I*b*). In the crystal of DIKSOG, mol­ecules are linked by pairs of N—H⋯N hydrogen bonds, forming inversion dimers, while in the crystal of KUTFAH, mol­ecules are linked into chains by N—H⋯N hydrogen bonds.

## Synthesis and crystallization   

The initial 4-phenyl-1,3-di­hydro-2*H*-benzo[*b*][1,4]diazepine-2-thio­nes (II*a*) and (II*b*) were synthesized from the corres­ponding 4-phenyl-1,3-di­hydro-2*H*-benzo[*b*][1,4]diazepin-2-ones according to the procedure described previously (Solomko *et al.*, 1990 ▸). The synthesis of the title compound (I*b*) is illustrated in Fig. 1 ▸.

**General procedure:**

Hydrazine hydrate (0.5 ml, 85% aq. solution) was added to a solution of the corresponding 4-phenyl-1,3-di­hydro-2*H*-benzo[*b*][1,4]diazepine-2-thio­nes, (II*a*) or (II*b*), (5 mmol) in ethanol (40 ml). The mixture was heated at reflux for 3 h (TLC monitoring), then the solvent and the excess of hydrazine hydrate were removed under reduced pressure. The residue was washed with small amounts of cold alcohol. Colourless crystals of (I*a*) and (I*b*) were grown by recrystallization of the crude product from ethanol solution.

**Spectroscopic and analytical data for (I** ***a*** **):**

Yield 0.91 g, 73%; m.p. 415–417 K [415–417 K from ethanol in accordance with Essassi & Salem (1985 ▸)]. IR *ν*_max_ (KBr): 3410–3220, 2970, 1605, 1545, 1505, 1260, 1030, 920, 860, 810 cm^−1^. ^1^H NMR (DMSO-*d*_6_, 400 MHz): *δ* 4.91 (*s*, 2H, NH_2_), 6.16 (*s*, 1H, CH), 6.40–6.79 (*m*, 3H, ArH + NH), 7.03–7.95 (*m*, 7H, ArH), 12.42 (*s*, 1H, NH) ppm. MS (EI) *m*/*z* (rel. intensity): 251 [*M* + H] (18), 250 [*M* ^+^] (100), 249 [*M* – H] (52), 234 (8), 233 (7), 221 (5), 219 (13), 132 (18), 131 (10), 130 (5), 125 (5), 119 (16), 104 (6), 103 (8), 102 (4), 92 (4), 91 (4), 77 (9). Analysis calculated for C_15_H_14_N_4_ (250.12): C, 71.98; H, 5.64; N, 22.38; found: C, 72.12; H, 5.54; N, 22.26.

**Spectroscopic and analytical data for (I** ***b*** **)**:

Yield 0.99 g, 70%; m.p. 468–470 K. IR *ν*_max_ (KBr): 3400–3210, 2975, 1600, 1560, 1500, 1250, 1145, 1000, 960, 920, 880, 855, 800 cm^−1^. ^1^H NMR (Solv, MHz): *δ* 4.95 (*s*, 2H, NH_2_), 6.27 (*s*, 1H, CH), 6.57–6.66 (*m*, 2H, ArH + NH), 7.30–7.79 (*m*, 7H, ArH), 12.49 (*s*, 1H, NH) ppm. MS (EI) *m*/*z* (rel. intensity): 285 [*M* + H] (34), 284 [*M* ^+^] (100), 283 [*M* – H] (44), 269 (6), 268 (10), 267 (12), 255 (8), 253 (12), 168 (8), 167 (8), 166 (25), 165 (13), 164 (7), 131 (7), 119 (26), 104 (8), 103 (7), 102 (7), 91 (6), 77 (13). Analysis calculated for C_15_H_13_ClN_4_ (284.08): C, 63.27; H, 4.60; N, 19.68; found: C, 63.08; H, 4.71; N, 19.73.

## Refinement   

Crystal data, data collection and structure refinement details are summarized in Table 2 ▸. All of the H atoms could be located from difference-Fourier maps. The C-bound H atoms were included in calculated positions and treated as riding: C—H = 0.93 Å with 1.2*U*_eq_(C). The N-bound H atoms were located in difference-Fourier maps and freely refined.

## Supplementary Material

Crystal structure: contains datablock(s) I, Global. DOI: 10.1107/S2056989017007381/su5369sup1.cif

Structure factors: contains datablock(s) I. DOI: 10.1107/S2056989017007381/su5369Isup2.hkl

Supporting information file. DOI: 10.1107/S2056989017007381/su5369Isup3.cml

CCDC reference: 703162

Additional supporting information:  crystallographic information; 3D view; checkCIF report

## Figures and Tables

**Table 1:** Hydrogen-bond geometry (Å, °) *Cg*3 is the centroid of the C10*A*–C15*A* ring.

*D*—H⋯*A*	*D*—H	H⋯*A*	*D*⋯*A*	*D*—H⋯*A*
N2*A*—H2*NA*⋯N4*B* ^i^	0.87 (2)	2.44 (2)	3.127 (3)	136 (2)
N3*A*—H3*NA*⋯N1*B* ^i^	0.82 (2)	2.17 (2)	2.973 (2)	168 (2)
N2*B*—H2*NB*⋯N4*A* ^i^	0.87 (2)	2.50 (2)	3.159 (3)	134 (2)
N3*B*—H3*NB*⋯N1*A* ^i^	0.83 (2)	2.20 (2)	3.019 (2)	169 (2)
N4*B*—H4*ND*⋯N1*A* ^ii^	0.89 (2)	2.43 (2)	3.207 (3)	146 (2)
C11*B*—H11*B*⋯*Cg*3^iii^	0.93	2.97	3.541 (2)	121

Symmetry codes: (i) 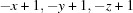; (ii) 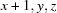; (iii) 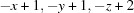.

**Table 2:** Experimental details

Crystal data	
Chemical formula	C_15_H_13_ClN_4_
*M*_r_	284.74
Crystal system, space group	Monoclinic, *P*2_1_/*c*
Temperature (K)	293
*a*, *b*, *c* (Å)	10.0709 (17), 20.322 (6), 13.886 (4)
β (°)	102.776 (18)
*V* (Å^3^)	2771.7 (12)
*Z*	8
Radiation type	Mo *K*α
μ (mm^−1^)	0.27
Crystal size (mm)	0.20 × 0.10 × 0.10

Data collection	
Diffractometer	Agilent Xcalibur Sapphire3
Absorption correction	Multi-scan (*CrysAlis RED*; Agilent, 2012 ▸).
*T*_min_, *T*_max_	0.649, 1.000
No. of measured, independent and observed [*I* > 2σ(*I*)] reflections	15157, 4795, 3132
*R*_int_	0.027
(sin θ/λ)_max_ (Å^−1^)	0.595

Refinement	
*R*[*F* ^2^ > 2σ(*F* ^2^)], *wR*(*F* ^2^), *S*	0.037, 0.102, 0.94
No. of reflections	4795
No. of parameters	393
H-atom treatment	H atoms treated by a mixture of independent and constrained refinement
Δρ_max_, Δρ_min_ (e Å^−3^)	0.16, −0.21

Computer programs: *CrysAlis CCD* and *CrysAlis RED* (Agilent, 2012 ▸), *SHELXS2014* (Sheldrick, 2008 ▸), *SHELXL2014* (Sheldrick, 2015 ▸), *Mercury* (Macrae *et al.*, 2008 ▸) and *PLATON* (Spek, 2009 ▸).

## supplementary crystallographic information

### Crystal data

**Table:**

C_15_H_13_ClN_4_	*F*(000) = 1184
*M**_r_* = 284.74	*D*_x_ = 1.365 Mg m^−^^3^
Monoclinic, *P*2_1_/*c*	Mo *K*α radiation, λ = 0.71073 Å
*a* = 10.0709 (17) Å	Cell parameters from 5031 reflections
*b* = 20.322 (6) Å	θ = 2.0–31.5°
*c* = 13.886 (4) Å	µ = 0.27 mm^−^^1^
β = 102.776 (18)°	*T* = 293 K
*V* = 2771.7 (12) Å^3^	Parallelepiped, colourless
*Z* = 8	0.20 × 0.10 × 0.10 mm

### Data collection

**Table:**

Agilent Xcalibur Sapphire3 diffractometer	4795 independent reflections
Radiation source: Enhance (Mo) X-ray Source	3132 reflections with *I* > 2σ(*I*)
Detector resolution: 16.1827 pixels mm^-1^	*R*_int_ = 0.027
ω–scan	θ_max_ = 25.0°, θ_min_ = 2.5°
Absorption correction: multi-scan (CrysAlis RED; Agilent, 2012).	*h* = −11→11
*T*_min_ = 0.649, *T*_max_ = 1.000	*k* = −24→24
15157 measured reflections	*l* = −16→15

### Refinement

**Table:**

Refinement on *F*^2^	Primary atom site location: structure-invariant direct methods
Least-squares matrix: full	Secondary atom site location: difference Fourier map
*R*[*F*^2^ > 2σ(*F*^2^)] = 0.037	Hydrogen site location: mixed
*wR*(*F*^2^) = 0.102	H atoms treated by a mixture of independent and constrained refinement
*S* = 0.94	*w* = 1/[σ^2^(*F*_o_^2^) + (0.064*P*)^2^] where *P* = (*F*_o_^2^ + 2*F*_c_^2^)/3
4795 reflections	(Δ/σ)_max_ = 0.001
393 parameters	Δρ_max_ = 0.16 e Å^−^^3^
0 restraints	Δρ_min_ = −0.21 e Å^−^^3^

### Special details

**Table:**

Geometry. Bond distances, angles etc. have been calculated using the rounded fractional coordinates. All su's are estimated from the variances of the (full) variance-covariance matrix. The cell esds are taken into account in the estimation of distances, angles and torsion angles

### Fractional atomic coordinates and isotropic or equivalent isotropic displacement parameters (Å^2^)

**Table:**

	*x*	*y*	*z*	*U*_iso_*/*U*_eq_	
Cl1A	0.49786 (7)	0.24353 (3)	0.83066 (4)	0.0730 (2)	
Cl1B	0.96923 (7)	0.24226 (3)	0.84367 (4)	0.0776 (2)	
N1A	0.21769 (16)	0.51489 (7)	0.60279 (10)	0.0457 (5)	
N2A	0.18787 (17)	0.55321 (8)	0.67697 (11)	0.0457 (5)	
N3A	0.39006 (18)	0.44024 (8)	0.58852 (12)	0.0492 (6)	
N4A	0.55691 (19)	0.39198 (9)	0.47173 (13)	0.0511 (6)	
C1A	0.27518 (18)	0.54304 (9)	0.76473 (12)	0.0415 (6)	
C2A	0.36726 (19)	0.49617 (9)	0.74824 (12)	0.0461 (6)	
C3A	0.32764 (18)	0.48054 (9)	0.64684 (12)	0.0411 (6)	
C4A	0.46130 (18)	0.38147 (8)	0.61844 (12)	0.0403 (6)	
C5A	0.54902 (18)	0.35744 (9)	0.55934 (13)	0.0421 (6)	
C6A	0.61960 (19)	0.29883 (9)	0.58605 (14)	0.0518 (7)	
C7A	0.6070 (2)	0.26396 (10)	0.66977 (15)	0.0580 (7)	
C8A	0.5203 (2)	0.28827 (9)	0.72658 (13)	0.0509 (7)	
C9A	0.44765 (19)	0.34626 (9)	0.70194 (12)	0.0460 (6)	
C10A	0.26315 (19)	0.57736 (8)	0.85694 (12)	0.0424 (6)	
C11A	0.3742 (2)	0.57789 (10)	0.93726 (13)	0.0539 (7)	
C12A	0.3656 (2)	0.60944 (11)	1.02456 (15)	0.0625 (8)	
C13A	0.2459 (2)	0.64019 (10)	1.03356 (15)	0.0594 (8)	
C14A	0.1350 (2)	0.63963 (10)	0.95502 (15)	0.0604 (8)	
C15A	0.1432 (2)	0.60866 (9)	0.86679 (14)	0.0533 (7)	
N1B	0.72238 (17)	0.52522 (8)	0.62179 (11)	0.0516 (5)	
N2B	0.69078 (18)	0.56217 (9)	0.69663 (11)	0.0513 (6)	
N3B	0.88215 (17)	0.44361 (8)	0.60966 (12)	0.0488 (6)	
N4B	1.04647 (18)	0.39335 (9)	0.49141 (13)	0.0510 (6)	
C1B	0.76350 (18)	0.54428 (9)	0.78701 (12)	0.0413 (6)	
C2B	0.84788 (18)	0.49384 (9)	0.77092 (12)	0.0451 (6)	
C3B	0.81946 (18)	0.48419 (9)	0.66735 (12)	0.0423 (6)	
C4B	0.94813 (18)	0.38342 (9)	0.63783 (12)	0.0420 (6)	
C5B	1.03456 (18)	0.35833 (9)	0.57797 (13)	0.0441 (6)	
C6B	1.0996 (2)	0.29824 (9)	0.60360 (14)	0.0543 (7)	
C7B	1.0828 (2)	0.26284 (10)	0.68562 (15)	0.0600 (8)	
C8B	0.9971 (2)	0.28790 (10)	0.74217 (14)	0.0539 (7)	
C9B	0.93023 (19)	0.34753 (9)	0.71968 (13)	0.0480 (6)	
C10B	0.75168 (17)	0.57770 (9)	0.87960 (12)	0.0403 (6)	
C11B	0.8117 (2)	0.54986 (10)	0.97074 (13)	0.0515 (7)	
C12B	0.8070 (2)	0.58188 (11)	1.05840 (14)	0.0563 (7)	
C13B	0.74198 (19)	0.64222 (10)	1.05668 (14)	0.0527 (7)	
C14B	0.6804 (2)	0.67007 (10)	0.96768 (15)	0.0573 (7)	
C15B	0.6852 (2)	0.63790 (9)	0.87961 (14)	0.0517 (7)	
H2NA	0.120 (2)	0.5806 (10)	0.6599 (14)	0.059 (6)*	
H3NA	0.3590 (18)	0.4435 (9)	0.5292 (13)	0.042 (5)*	
H2A	0.44000	0.47860	0.79430	0.0550*	
H4NB	0.631 (2)	0.3782 (9)	0.4504 (15)	0.058 (6)*	
H4NA	0.563 (2)	0.4346 (12)	0.4845 (16)	0.076 (7)*	
H6A	0.67640	0.28270	0.54700	0.0620*	
H7A	0.65550	0.22520	0.68740	0.0700*	
H9A	0.39010	0.36150	0.74100	0.0550*	
H11A	0.45470	0.55700	0.93240	0.0650*	
H12A	0.44070	0.60990	1.07740	0.0750*	
H13A	0.24060	0.66100	1.09220	0.0710*	
H14A	0.05440	0.66000	0.96080	0.0720*	
H15A	0.06800	0.60880	0.81400	0.0640*	
H2B	0.91050	0.47090	0.81830	0.0540*	
H2NB	0.624 (2)	0.5897 (10)	0.6809 (15)	0.059 (6)*	
H3NB	0.8595 (18)	0.4500 (9)	0.5492 (14)	0.045 (5)*	
H6B	1.15590	0.28140	0.56470	0.0650*	
H4ND	1.063 (2)	0.4357 (11)	0.5059 (15)	0.065 (7)*	
H7B	1.12810	0.22320	0.70220	0.0720*	
H4NC	1.116 (2)	0.3777 (10)	0.4691 (15)	0.062 (6)*	
H9B	0.87370	0.36350	0.75900	0.0580*	
H11B	0.85550	0.50940	0.97280	0.0620*	
H12B	0.84760	0.56270	1.11850	0.0680*	
H13B	0.74000	0.66370	1.11550	0.0630*	
H14B	0.63570	0.71020	0.96620	0.0690*	
H15B	0.64320	0.65700	0.81980	0.0620*	

### Atomic displacement parameters (Å^2^)

**Table:**

	*U*^11^	*U*^22^	*U*^33^	*U*^12^	*U*^13^	*U*^23^
Cl1A	0.1159 (5)	0.0493 (3)	0.0465 (3)	−0.0072 (3)	0.0021 (3)	0.0085 (2)
Cl1B	0.1147 (5)	0.0600 (4)	0.0563 (3)	0.0078 (3)	0.0148 (3)	0.0152 (3)
N1A	0.0554 (9)	0.0491 (9)	0.0332 (8)	0.0080 (8)	0.0110 (7)	−0.0007 (7)
N2A	0.0530 (10)	0.0473 (9)	0.0363 (8)	0.0095 (9)	0.0087 (8)	−0.0029 (7)
N3A	0.0674 (11)	0.0510 (10)	0.0297 (8)	0.0155 (9)	0.0120 (8)	0.0032 (7)
N4A	0.0593 (11)	0.0478 (11)	0.0510 (10)	0.0010 (9)	0.0224 (9)	−0.0045 (8)
C1A	0.0506 (11)	0.0394 (10)	0.0353 (9)	−0.0022 (9)	0.0110 (9)	0.0019 (8)
C2A	0.0547 (11)	0.0487 (11)	0.0330 (9)	0.0085 (10)	0.0059 (9)	0.0024 (8)
C3A	0.0503 (11)	0.0394 (10)	0.0349 (9)	0.0028 (9)	0.0122 (9)	0.0033 (8)
C4A	0.0456 (10)	0.0374 (10)	0.0349 (9)	0.0002 (9)	0.0025 (8)	−0.0057 (8)
C5A	0.0448 (10)	0.0397 (10)	0.0403 (10)	−0.0049 (9)	0.0065 (8)	−0.0070 (8)
C6A	0.0520 (12)	0.0440 (11)	0.0577 (12)	0.0048 (10)	0.0085 (10)	−0.0110 (10)
C7A	0.0652 (14)	0.0389 (11)	0.0615 (13)	0.0079 (11)	−0.0037 (11)	−0.0020 (10)
C8A	0.0666 (13)	0.0386 (11)	0.0404 (10)	−0.0064 (10)	−0.0032 (10)	−0.0010 (8)
C9A	0.0557 (11)	0.0439 (11)	0.0363 (10)	−0.0007 (10)	0.0059 (9)	−0.0035 (8)
C10A	0.0543 (11)	0.0378 (10)	0.0365 (9)	−0.0060 (9)	0.0130 (9)	−0.0006 (8)
C11A	0.0550 (12)	0.0610 (13)	0.0452 (11)	−0.0021 (11)	0.0103 (10)	−0.0064 (10)
C12A	0.0704 (14)	0.0710 (14)	0.0434 (12)	−0.0096 (12)	0.0070 (11)	−0.0121 (10)
C13A	0.0803 (15)	0.0569 (13)	0.0451 (12)	−0.0094 (12)	0.0224 (12)	−0.0136 (10)
C14A	0.0703 (14)	0.0598 (13)	0.0566 (13)	0.0066 (12)	0.0261 (12)	−0.0080 (11)
C15A	0.0580 (12)	0.0562 (12)	0.0448 (11)	0.0044 (11)	0.0096 (10)	−0.0028 (9)
N1B	0.0612 (10)	0.0596 (10)	0.0348 (8)	0.0172 (9)	0.0124 (8)	0.0032 (7)
N2B	0.0591 (11)	0.0596 (11)	0.0360 (9)	0.0234 (9)	0.0123 (8)	0.0060 (8)
N3B	0.0626 (11)	0.0525 (10)	0.0327 (8)	0.0131 (8)	0.0135 (8)	0.0021 (8)
N4B	0.0554 (11)	0.0484 (11)	0.0526 (10)	0.0004 (9)	0.0193 (9)	−0.0085 (8)
C1B	0.0440 (10)	0.0442 (10)	0.0359 (9)	0.0008 (9)	0.0090 (8)	0.0053 (8)
C2B	0.0483 (11)	0.0489 (11)	0.0360 (10)	0.0101 (9)	0.0047 (8)	0.0016 (8)
C3B	0.0456 (11)	0.0437 (10)	0.0384 (10)	0.0034 (9)	0.0112 (9)	0.0040 (8)
C4B	0.0437 (10)	0.0413 (10)	0.0373 (10)	0.0005 (9)	0.0010 (8)	−0.0048 (8)
C5B	0.0446 (10)	0.0452 (11)	0.0408 (10)	−0.0032 (9)	0.0061 (8)	−0.0102 (9)
C6B	0.0586 (12)	0.0474 (12)	0.0565 (12)	0.0068 (10)	0.0117 (10)	−0.0089 (10)
C7B	0.0703 (14)	0.0457 (12)	0.0584 (13)	0.0114 (11)	0.0020 (11)	−0.0052 (10)
C8B	0.0671 (13)	0.0460 (12)	0.0435 (10)	−0.0013 (11)	0.0016 (10)	−0.0017 (9)
C9B	0.0549 (12)	0.0472 (11)	0.0401 (10)	0.0027 (10)	0.0066 (9)	−0.0033 (9)
C10B	0.0416 (10)	0.0427 (10)	0.0380 (9)	−0.0035 (9)	0.0119 (8)	0.0025 (8)
C11B	0.0607 (12)	0.0507 (12)	0.0423 (11)	0.0063 (10)	0.0096 (10)	0.0021 (9)
C12B	0.0603 (13)	0.0687 (14)	0.0383 (10)	−0.0018 (12)	0.0073 (10)	0.0015 (10)
C13B	0.0583 (12)	0.0567 (12)	0.0459 (11)	−0.0104 (11)	0.0178 (10)	−0.0134 (10)
C14B	0.0655 (13)	0.0531 (12)	0.0565 (13)	0.0059 (11)	0.0206 (11)	−0.0018 (10)
C15B	0.0596 (12)	0.0520 (12)	0.0451 (11)	0.0099 (10)	0.0148 (10)	0.0075 (9)

### Geometric parameters (Å, º)

**Table:**

Cl1A—C8A	1.764 (2)	C7A—H7A	0.9300
Cl1B—C8B	1.761 (2)	C9A—H9A	0.9300
N1A—C3A	1.337 (2)	C11A—H11A	0.9300
N1A—N2A	1.376 (2)	C12A—H12A	0.9300
N2A—C1A	1.352 (2)	C13A—H13A	0.9300
N3A—C4A	1.408 (2)	C14A—H14A	0.9300
N3A—C3A	1.395 (2)	C15A—H15A	0.9300
N4A—C5A	1.422 (3)	C1B—C2B	1.381 (3)
C1A—C10A	1.486 (2)	C1B—C10B	1.482 (2)
C1A—C2A	1.383 (3)	C2B—C3B	1.417 (2)
C2A—C3A	1.412 (2)	N2B—H2NB	0.87 (2)
N2A—H2NA	0.87 (2)	N3B—H3NB	0.830 (19)
N3A—H3NA	0.817 (18)	C4B—C9B	1.395 (3)
C4A—C9A	1.395 (2)	C4B—C5B	1.424 (3)
N4A—H4NB	0.91 (2)	N4B—H4ND	0.89 (2)
N4A—H4NA	0.88 (2)	N4B—H4NC	0.89 (2)
C4A—C5A	1.419 (3)	C5B—C6B	1.394 (3)
C5A—C6A	1.395 (3)	C6B—C7B	1.389 (3)
C6A—C7A	1.391 (3)	C7B—C8B	1.386 (3)
C7A—C8A	1.391 (3)	C8B—C9B	1.388 (3)
C8A—C9A	1.389 (3)	C10B—C11B	1.396 (3)
C10A—C15A	1.398 (3)	C10B—C15B	1.395 (3)
C10A—C11A	1.394 (3)	C11B—C12B	1.390 (3)
C11A—C12A	1.391 (3)	C12B—C13B	1.388 (3)
C12A—C13A	1.388 (3)	C13B—C14B	1.376 (3)
C13A—C14A	1.378 (3)	C14B—C15B	1.397 (3)
C14A—C15A	1.396 (3)	C2B—H2B	0.9300
N1B—N2B	1.375 (2)	C6B—H6B	0.9300
N1B—C3B	1.333 (2)	C7B—H7B	0.9300
C2A—H2A	0.9300	C9B—H9B	0.9300
N2B—C1B	1.355 (2)	C11B—H11B	0.9300
N3B—C4B	1.406 (2)	C12B—H12B	0.9300
N3B—C3B	1.394 (2)	C13B—H13B	0.9300
N4B—C5B	1.425 (3)	C14B—H14B	0.9300
C6A—H6A	0.9300	C15B—H15B	0.9300
			
N2A—N1A—C3A	104.40 (14)	C10A—C15A—H15A	120.00
N1A—N2A—C1A	112.47 (15)	C14A—C15A—H15A	120.00
C3A—N3A—C4A	126.32 (15)	N2B—C1B—C2B	105.97 (15)
N2A—C1A—C2A	106.40 (15)	N2B—C1B—C10B	123.35 (17)
N2A—C1A—C10A	122.97 (16)	C2B—C1B—C10B	130.61 (16)
C2A—C1A—C10A	130.62 (16)	C1B—C2B—C3B	105.88 (15)
C1A—C2A—C3A	105.54 (16)	N1B—N2B—H2NB	117.3 (14)
N1A—N2A—H2NA	116.4 (13)	C1B—N2B—H2NB	129.7 (14)
C1A—N2A—H2NA	131.1 (13)	N1B—C3B—N3B	118.36 (15)
N1A—C3A—N3A	118.34 (15)	C3B—N3B—C4B	126.95 (16)
C3A—N3A—H3NA	114.6 (13)	C3B—N3B—H3NB	115.5 (13)
C4A—N3A—H3NA	115.0 (13)	C4B—N3B—H3NB	115.1 (13)
N1A—C3A—C2A	111.19 (16)	N1B—C3B—C2B	110.95 (16)
N3A—C3A—C2A	130.28 (17)	N3B—C3B—C2B	130.52 (17)
H4NB—N4A—H4NA	110.1 (18)	H4ND—N4B—H4NC	107.7 (19)
C5A—C4A—C9A	119.52 (16)	C5B—C4B—C9B	119.60 (17)
C5A—N4A—H4NA	109.1 (14)	C5B—N4B—H4NC	109.7 (13)
N3A—C4A—C5A	117.59 (15)	N3B—C4B—C5B	117.48 (16)
N3A—C4A—C9A	122.89 (16)	N3B—C4B—C9B	122.91 (17)
C5A—N4A—H4NB	109.5 (13)	C5B—N4B—H4ND	109.8 (13)
N4A—C5A—C4A	119.00 (16)	N4B—C5B—C4B	119.34 (16)
N4A—C5A—C6A	121.84 (17)	N4B—C5B—C6B	122.04 (17)
C4A—C5A—C6A	119.08 (16)	C4B—C5B—C6B	118.54 (17)
C5A—C6A—C7A	121.47 (18)	C5B—C6B—C7B	121.84 (18)
C6A—C7A—C8A	118.55 (18)	C6B—C7B—C8B	118.58 (19)
C7A—C8A—C9A	121.61 (17)	C7B—C8B—C9B	121.69 (18)
Cl1A—C8A—C7A	119.48 (15)	Cl1B—C8B—C7B	119.31 (16)
Cl1A—C8A—C9A	118.89 (15)	Cl1B—C8B—C9B	118.99 (15)
C4A—C9A—C8A	119.77 (17)	C4B—C9B—C8B	119.74 (17)
C1A—C10A—C11A	119.28 (17)	C1B—C10B—C11B	119.94 (17)
C1A—C10A—C15A	122.31 (16)	C1B—C10B—C15B	122.19 (16)
C11A—C10A—C15A	118.41 (16)	C11B—C10B—C15B	117.84 (16)
C10A—C11A—C12A	120.49 (19)	C10B—C11B—C12B	120.82 (19)
C11A—C12A—C13A	120.58 (19)	C11B—C12B—C13B	120.38 (18)
C12A—C13A—C14A	119.55 (19)	C12B—C13B—C14B	119.74 (18)
C13A—C14A—C15A	120.23 (19)	C13B—C14B—C15B	119.86 (19)
C10A—C15A—C14A	120.73 (18)	C10B—C15B—C14B	121.34 (17)
N2B—N1B—C3B	104.53 (14)	C1B—C2B—H2B	127.00
C3A—C2A—H2A	127.00	C3B—C2B—H2B	127.00
C1A—C2A—H2A	127.00	C5B—C6B—H6B	119.00
N1B—N2B—C1B	112.66 (16)	C7B—C6B—H6B	119.00
C3B—N3B—C4B	126.95 (16)	C6B—C7B—H7B	121.00
C5A—C6A—H6A	119.00	C8B—C7B—H7B	121.00
C7A—C6A—H6A	119.00	C4B—C9B—H9B	120.00
C8A—C7A—H7A	121.00	C8B—C9B—H9B	120.00
C6A—C7A—H7A	121.00	C10B—C11B—H11B	120.00
C8A—C9A—H9A	120.00	C12B—C11B—H11B	120.00
C4A—C9A—H9A	120.00	C11B—C12B—H12B	120.00
C10A—C11A—H11A	120.00	C13B—C12B—H12B	120.00
C12A—C11A—H11A	120.00	C12B—C13B—H13B	120.00
C11A—C12A—H12A	120.00	C14B—C13B—H13B	120.00
C13A—C12A—H12A	120.00	C13B—C14B—H14B	120.00
C14A—C13A—H13A	120.00	C15B—C14B—H14B	120.00
C12A—C13A—H13A	120.00	C10B—C15B—H15B	119.00
C13A—C14A—H14A	120.00	C14B—C15B—H15B	119.00
C15A—C14A—H14A	120.00		
			
C3A—N1A—N2A—C1A	−0.5 (2)	C3B—N1B—N2B—C1B	−1.2 (2)
N2A—N1A—C3A—N3A	−175.01 (16)	N2B—N1B—C3B—N3B	−174.38 (17)
N2A—N1A—C3A—C2A	0.5 (2)	N2B—N1B—C3B—C2B	1.2 (2)
N1A—N2A—C1A—C2A	0.2 (2)	N1B—N2B—C1B—C2B	0.7 (2)
N1A—N2A—C1A—C10A	−178.67 (16)	N1B—N2B—C1B—C10B	178.09 (17)
C4A—N3A—C3A—N1A	−149.84 (18)	C4B—N3B—C3B—N1B	−156.03 (18)
C4A—N3A—C3A—C2A	35.7 (3)	C4B—N3B—C3B—C2B	29.4 (3)
C3A—N3A—C4A—C5A	−162.61 (18)	C3B—N3B—C4B—C5B	−164.28 (18)
C3A—N3A—C4A—C9A	18.2 (3)	C3B—N3B—C4B—C9B	16.9 (3)
N2A—C1A—C2A—C3A	0.1 (2)	N2B—C1B—C2B—C3B	0.1 (2)
C10A—C1A—C2A—C3A	178.87 (19)	C10B—C1B—C2B—C3B	−177.05 (19)
N2A—C1A—C10A—C11A	−163.29 (18)	N2B—C1B—C10B—C11B	169.08 (19)
N2A—C1A—C10A—C15A	17.6 (3)	N2B—C1B—C10B—C15B	−12.8 (3)
C2A—C1A—C10A—C11A	18.1 (3)	C2B—C1B—C10B—C11B	−14.3 (3)
C2A—C1A—C10A—C15A	−161.0 (2)	C2B—C1B—C10B—C15B	163.8 (2)
C1A—C2A—C3A—N1A	−0.4 (2)	C1B—C2B—C3B—N1B	−0.8 (2)
C1A—C2A—C3A—N3A	174.43 (19)	C1B—C2B—C3B—N3B	174.08 (19)
N3A—C4A—C5A—N4A	−2.4 (3)	N3B—C4B—C5B—N4B	−2.3 (3)
N3A—C4A—C5A—C6A	−179.06 (17)	N3B—C4B—C5B—C6B	−179.09 (17)
C9A—C4A—C5A—N4A	176.89 (17)	C9B—C4B—C5B—N4B	176.51 (17)
C9A—C4A—C5A—C6A	0.2 (3)	C9B—C4B—C5B—C6B	−0.3 (3)
N3A—C4A—C9A—C8A	179.43 (17)	N3B—C4B—C9B—C8B	178.98 (18)
C5A—C4A—C9A—C8A	0.2 (3)	C5B—C4B—C9B—C8B	0.2 (3)
N4A—C5A—C6A—C7A	−177.36 (18)	N4B—C5B—C6B—C7B	−177.05 (19)
C4A—C5A—C6A—C7A	−0.7 (3)	C4B—C5B—C6B—C7B	−0.4 (3)
C5A—C6A—C7A—C8A	0.9 (3)	C5B—C6B—C7B—C8B	1.0 (3)
C6A—C7A—C8A—Cl1A	177.79 (15)	C6B—C7B—C8B—Cl1B	177.56 (16)
C6A—C7A—C8A—C9A	−0.4 (3)	C6B—C7B—C8B—C9B	−1.1 (3)
Cl1A—C8A—C9A—C4A	−178.35 (14)	Cl1B—C8B—C9B—C4B	−178.18 (15)
C7A—C8A—C9A—C4A	−0.1 (3)	C7B—C8B—C9B—C4B	0.5 (3)
C1A—C10A—C11A—C12A	−179.78 (18)	C1B—C10B—C11B—C12B	177.10 (18)
C15A—C10A—C11A—C12A	−0.6 (3)	C15B—C10B—C11B—C12B	−1.1 (3)
C1A—C10A—C15A—C14A	179.10 (17)	C1B—C10B—C15B—C14B	−177.09 (18)
C11A—C10A—C15A—C14A	−0.1 (3)	C11B—C10B—C15B—C14B	1.0 (3)
C10A—C11A—C12A—C13A	0.8 (3)	C10B—C11B—C12B—C13B	0.2 (3)
C11A—C12A—C13A—C14A	−0.3 (3)	C11B—C12B—C13B—C14B	0.8 (3)
C12A—C13A—C14A—C15A	−0.4 (3)	C12B—C13B—C14B—C15B	−0.8 (3)
C13A—C14A—C15A—C10A	0.5 (3)	C13B—C14B—C15B—C10B	−0.1 (3)

### Hydrogen-bond geometry (Å, º)

Cg3 is the centroid of the C10*A*–C15*A* ring.

**Table:**

*D*—H···*A*	*D*—H	H···*A*	*D*···*A*	*D*—H···*A*
N2*A*—H2*NA*···N4*B*^i^	0.87 (2)	2.44 (2)	3.127 (3)	136 (2)
N3*A*—H3*NA*···N1*B*^i^	0.82 (2)	2.17 (2)	2.973 (2)	168 (2)
N2*B*—H2*NB*···N4*A*^i^	0.87 (2)	2.50 (2)	3.159 (3)	134 (2)
N3*B*—H3*NB*···N1*A*^i^	0.83 (2)	2.20 (2)	3.019 (2)	169 (2)
N4*B*—H4*ND*···N1*A*^ii^	0.89 (2)	2.43 (2)	3.207 (3)	146 (2)
C11*B*—H11*B*···*Cg*3^iii^	0.93	2.97	3.541 (2)	121

Symmetry codes: (i) −*x*+1, −*y*+1, −*z*+1; (ii) *x*+1, *y*, *z*; (iii) −*x*+1, −*y*+1, −*z*+2.
